# Endodontic and Orthodontic Management of Traumatically Intruded Teeth with Horizontal Root Fracture: A Case Report

**DOI:** 10.1155/2011/250267

**Published:** 2011-09-21

**Authors:** Marcos Jacobovitz, Adriana Maria Bonadio Lopes Ramos, Regina Karla de Pontes Lima, Fernanda Geraldes Pappen, Anna B. Fuks

**Affiliations:** ^1^Associação Paulista dos Cirurgiões Dentista Regional de São Carlos, São Carlos, SP, Brazil; ^2^Associação Paulista dos Cirurgiões Dentistas de Araraquara, São Paulo, SP, Brazil; ^3^Department of Endodontics, Faculdade de Odontologia de Araraquara, Universidade Estadual Paulista (UNESP), 14801-903 São Paulo, SP, Brazil; ^4^Department of Endodontics, Universidade Federal de Pelotas (UFPel), 96001-970 Pelotas, RS, Brazil; ^5^Department of Pediatric Dentistry, Hadassah School of Dental Medicine, Hebrew University of Jerusalem, Jerusalem 91120, Israel

## Abstract

This report describes the case of an 8-year-old boy that was referred to endodontic and orthodontic treatment after suffering an injury that led to intrusion of the maxillary right permanent central incisor, palatal displacement of the upper right lateral incisor, and root fracture of both central incisors. Both injured teeth were immature and had open apices. The intruded tooth was repositioned by endodontic and orthodontic management. Endodontic therapy was performed in both teeth with periodical changes of calcium-hydroxide-based paste as root canal dressing and introduction of MTA as an apical barrier. The postoperative course was uneventful with clinical and radiographic success up to 3 and 1/2 years of follow up. In the present case, orthodontic repositioning combined with endodontic therapy constitued a viable alternative treatment for intrusive luxations in immature permanent teeth.

## 1. Introduction

Traumatic intrusive luxation is one of the most severe forms of dental injuries, usually affecting the maxillary incisors. Intrusive luxation is characterized by the forced axial dislodgement of the tooth into its socket toward the alveolar bone, causing damage to the pulp, to the supporting structures, and to the neurovascular bundle and is often related to impacted fracture of the alveolar bone [1].

The consequence of such an occurrence, is a high risk of healing complications such as pulp necrosis, external inflammatory resorption, and external replacement resorption (ankylosis) [2]. 

In a study evaluating traumatized permanent incisors, Andreasen [3] found that when intrusion occurred in teeth with closed apices, pulp necrosis was present in 100% of the cases whereas for teeth with open apices this rate decreased to 62.5%. Thus, in cases of closed apices, endodontic intervention should be initiated as early as possible, to prevent inflammatory external root resorption.

Depending on the severity of the injury, different clinical approaches for treatment of intrusive luxation may be used:

- waiting for passive repositioning,
- surgical management [4],
- active repositioning by means of orthodontic traction.

Despite the variety of treatment modalities, rehabilitation of intruded teeth is always a challenge and a multidisciplinary approach is important to achieve a successful result.

The present clinical report shows a successful multidisciplinary treatment of both maxillary central incisors in which one of them had suffered a severe intrusive luxation. Complete healing was noticed by means of follow-up radiographs throughout 3 and 1/2 years. However, the use of mineral trioxide aggregate led to a mild grey discoloration of the crown.

## 2. Case Presentation

**Figure 1:**
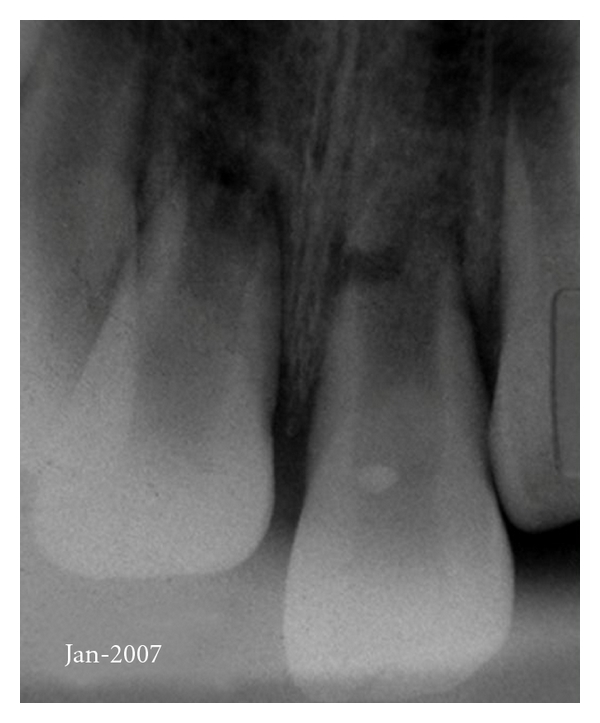
Preoperative radiograph. Severe intrusive luxation of tooth 11 and root fractures of apical thirds of both maxillary central incisors.

On January 2007, an 8-year-old boy suffered an injury in a playground see-saw affecting the region of maxillary anterior teeth. His parents sought for professional care in a pediatric dentistry clinic 5 days after the accident. At this occasion, a severe intrusive luxation of tooth 11 was diagnosed (more than 6 mm of intrusion from the incisal line (according to the classification of the Royal College of Surgeons of England [5]). In addition, root fractures of the apical third of both maxillary central incisors were also noticed in the radiograph (Figure 1). The patient was referred to both specialized endodontic and orthodontic care.

**Figure 2:**
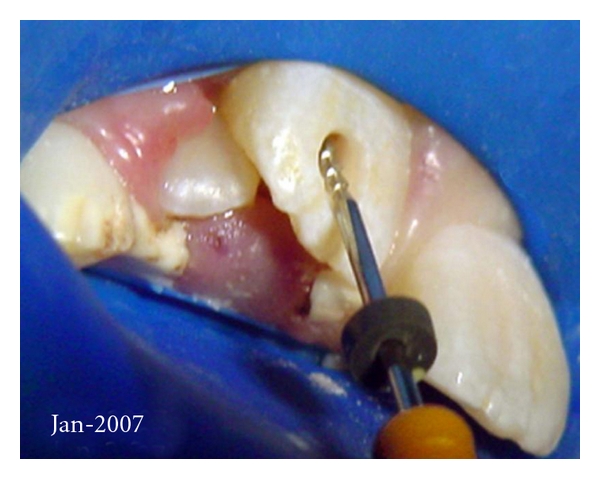
Buccal access for endodontic management.

Fifteen days later, specialized endodontic treatment was initiated. The placement of a rubber dam was adapted to the case, and due to the impossibility of conventional access form, the palatal surface, coronal access was carried out via buccal surface of the crown (Figure 2). Before the coronal access of the teeth, a vitality pulpal test was performed using Endo Ice (Hygenic-Akron OH, USA), and at that moment, no sensitivity was related by the patient. In order to ensure a safe access in supposed pulpless teeth, no anesthesia was applied. The access on 11 was performed successfully in this context, whereas on 21, a mild sensitivity was related during the access with the bur, so we proceeded with anesthesia with 1/2 tube of mepivacaine 3%, without vasoconstrictor. After this step, the diagnosis of necrotic pulp in both teeth was confirmed: neither bleeding nor a consistent pulp was found during the root canal preparation. A manual instrumentation and the cleansing of the root canals were performed (cleaning and shaping) with constant irrigation using preheated 1% sodium hypochlorite. 

The measurement of the working length was made by the Ingle method, and it was determined according Andreasen [6] for fractured roots. After endodontic instrumentation, the root canals were dried by means of capillary tips (Ultradent Inc. South Jordan, Utah, USA), attached to a suction unit and a solution of buffered 14% ethylene diamine tetraacetic acid (Odhacam/Dentsply, Rio de Janeiro, RJ, Brazil) was applied into their pulpal chambers for 3 min under manual instrument agitation. Afterwards, a calcium hydroxide Ca(OH)_2_ paste dressing (Calen, SSW Artigos Dentários Ltda., Rio de Janeiro, RJ, Brazil) was placed into the root canals for 15 days aiming to alkalinize the environment and aid on removal of the remaining pulp tissue.

After the Ca(OH)_2_ paste removal, white MTA (Angelus, Londrina, BR, Brazil) was chosen for root canal filling. The material was placed into the root canals in half of the working length with the aid of an amalgam carrier and a manual plugger, resulting in a 3-mm apical plug. Calcium hydroxide dressing filled the portion of the root canal without MTA with periodical changes until the complete filling of root canal with gutta-pecha points and AH-Plus (Dentsply, DeTrey, Konstanz, Germany). During the treatment, between dressing changes, we rerestored the crowns of the teeth provisionally with Coltosol (Coltène Whaledent, Altstätten, Switzerland). 

**Figure 3:**
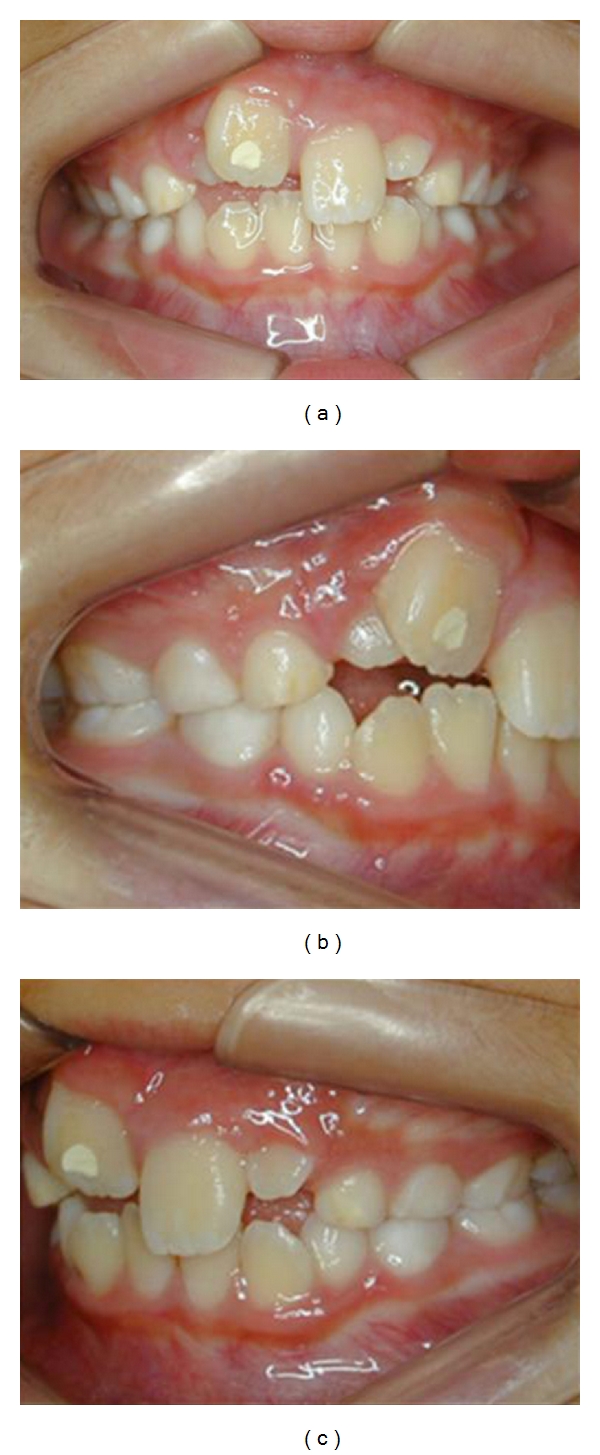
Intrusion and retroclination of tooth 11 and lingual rotation and displacement of tooth 12 (Jan. 2007).

**Figure 4:**
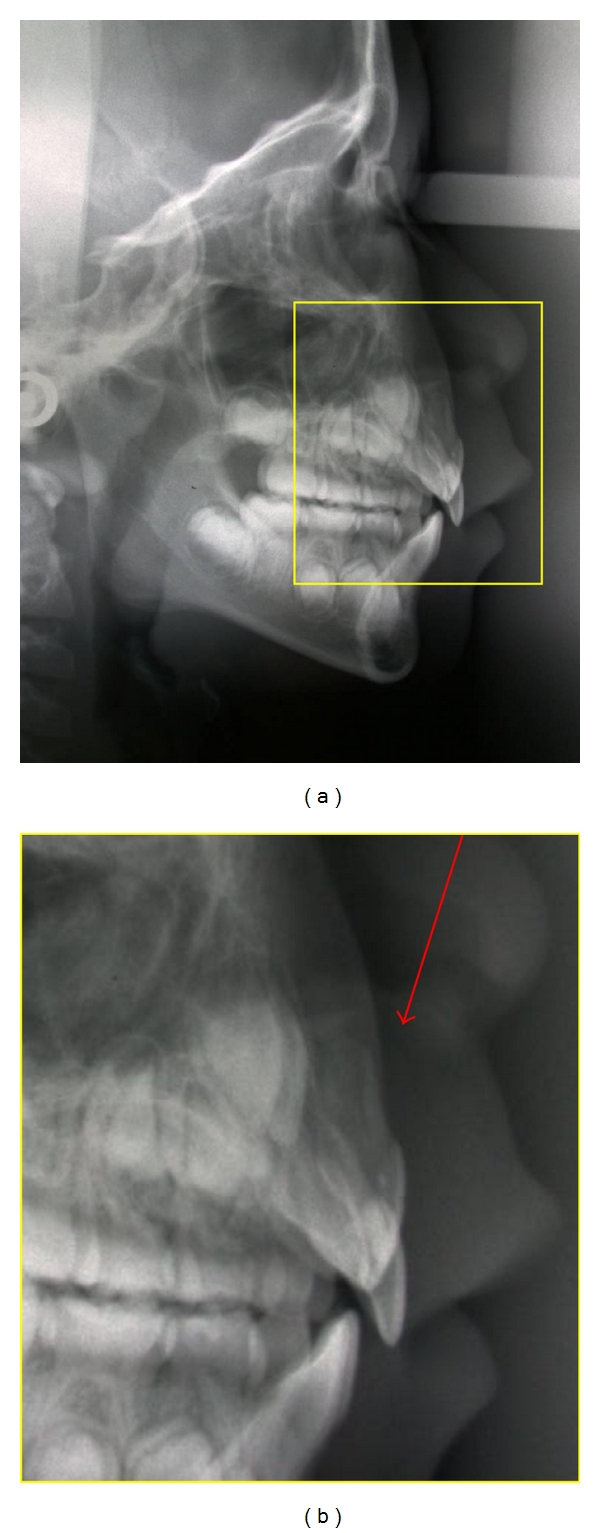
The risks of buccal osseous fenestration.

**Figure 5:**
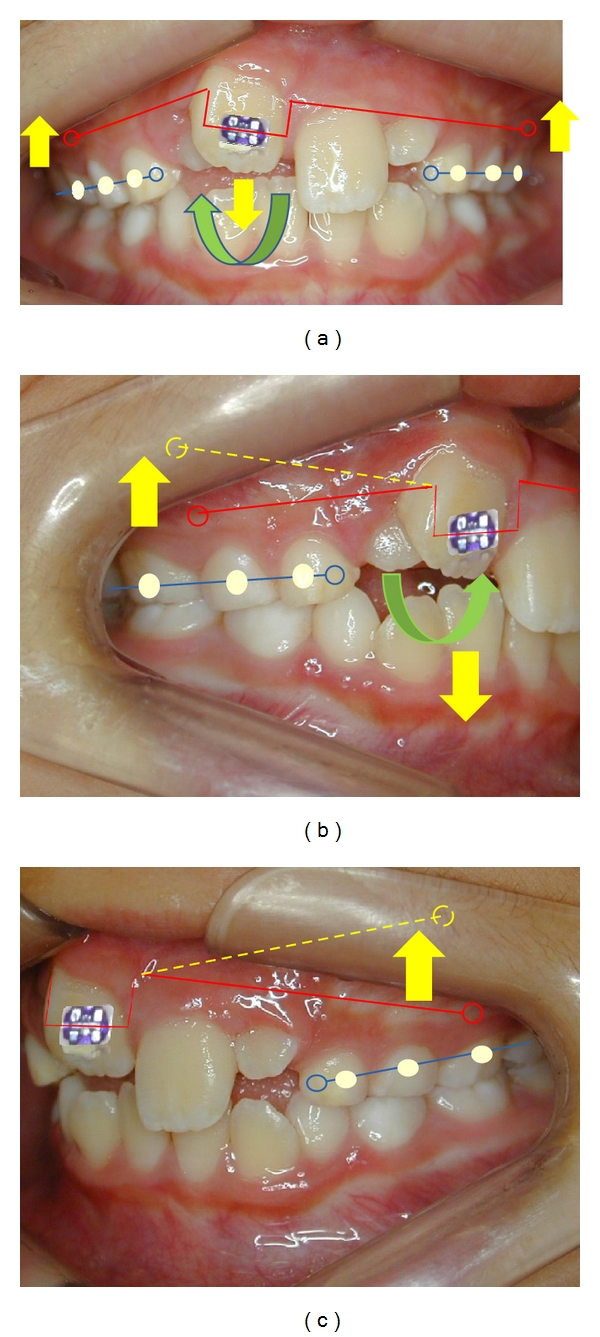
The torquing arch mechanics.

During the orthodontic management, the following characteristics were noticed due to trauma: intrusion and retroclination of tooth 11 and lingual rotation and displacement of tooth 12 (Figure 3). No space for the incisors alignment was yet available. Otherwise, in both lateral views, no evidence of abnormal changes in posterior occlusion was noticed for the patient age group. Considering the risks for repositioning the intruded teeth, such as buccal osseous fenestration (Figure 4), the segmented arch technique was chosen, aiming the control of exact applied forces and exact direction on active unit for the teeth repositioning, by means of a torching arch [7] (Figure 5). Moreover, to recover the lost space for the incisors realignment, a Haas palatal expansion appliance was utilized and built in the same orthodontic appliance [8]. The duration of orthodontic management was 7 months.

Since the initial visit and during the orthodontic management, the patient was invited for periodical follow-up visits in order to confirm the healing process. 

**Figure 6:**
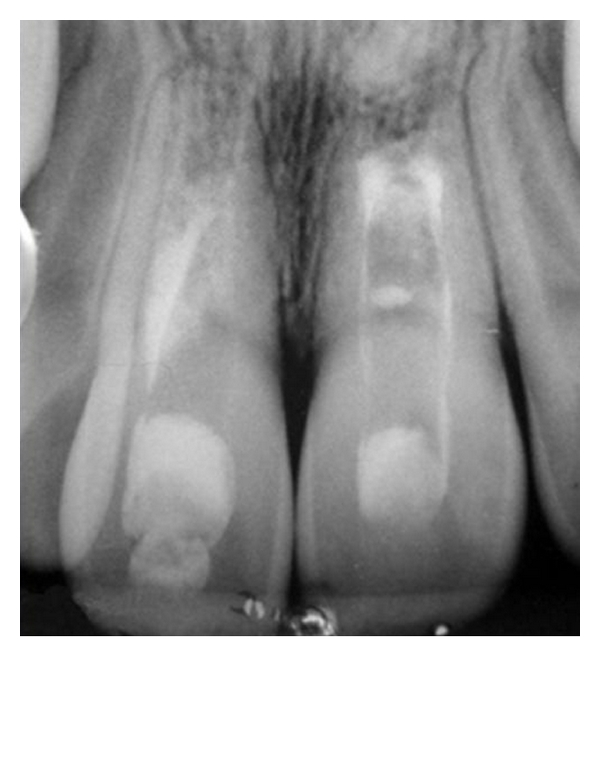
Twenty-month posttreatment radiograph (Sep. 2008).

**Figure 7:**
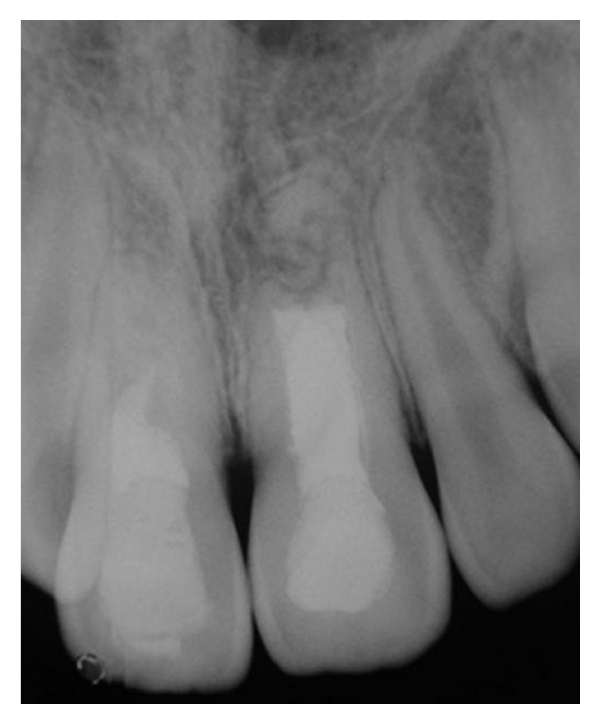
Three and half years posttreatment radiograph (June 2010).

**Figure 8:**
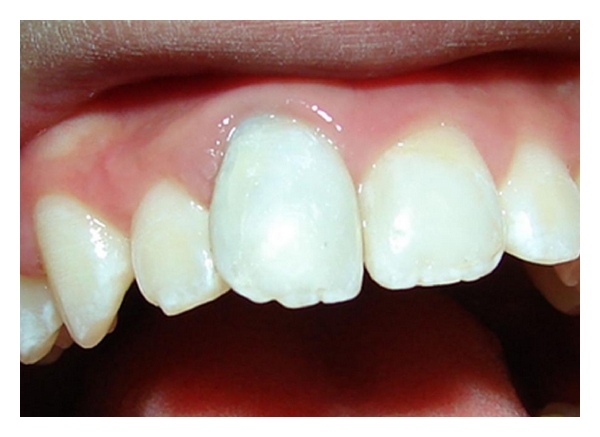
Posttreatment clinical aspect after 3 and 1/2 years, showing mild grey discoloration of the crown (June 2010).

The successful result of this case was verified by the absence of clinical symptoms and by follow-up radiographs after 3 and 1/2 years (Figures 6, 7, and 8).

## 3. Discussion

Luxative intrusion is a serious kind of injury of maxillary incisors and such an occurrence is found to be most frequent between 6 and 12 years of age and generally affecting 1.9% of traumatic injuries involving permanent teeth [9].

It is well established in the literature that pulpal necrosis will appear in permanent teeth with open apices that suffer from this kind of trauma [3, 10]. Throughout a 20-year-study period (from 1983 to 2003), no general agreement existed concerning the best treatment for intruded permanent teeth. The strategies for these kind of treatments must be planned according to the degree of severity of the injury [2].

The present clinical case shows a severe traumatic intrusive luxation according to the Royal College of Surgeons of England (intruded more than 6 mm). As complicating factors, the root of 11 was led to a buccal positioning, and its crown moved toward palatal face. Apical thirds of both roots were broken in the injury, and the Endodontic access in tooth 11 via palatal face was quite impossible, due to the depth of the intrusion and the close relationship with its neighbor, tooth 12.

Considering the degree of severity of the injury, different clinical modalities for intrusive luxation treatment may be used: passive repositioning, surgical management, and active repositioning by means of orthodontic traction. The main goal of these modalities of treatment is to achieve a guided repositioning of the tooth without the complications related to this injury. 

In the majority of the cases, the treatment of choice for traumatically intruded permanent teeth with complete root formation should be the orthodontic repositioning rather than the surgical repositioning [11].

As intruded teeth are at risk of replacement resorption, treatment with low risk of this complication should be defined as the best method. Accordingly, little information exists as to the possible influence of treatment on the development of the most severe complication following trauma [2].

Allowing for spontaneous re-eruption is the treatment of choice [12, 13] especially when the tooth apex is incomplete, according to the Royal College of Surgeons of England (RCSE), for cases on which the amount of intrusion is smaller than 3.0 mm, because, their high potential for eruption and pulpal or periodontal repair. This reeruption occurs especially when the dental pulp is vital, and hardly occurs when pulp necrosis is established [14]. So, in the present case, due to the characteristic of severe intrusion (more than 6 mm) and pulpal necrosis, the most indicated procedure was the multidisciplinary endodontic and orthodontic management. Orthodontic repositioning represent a more biological procedure for teeth who suffered this kind of injury [15] moreover, an emergency endodontic treatment prevents inflammatory root resorption [13, 15–17].

Due to the difficulty for root canal access in cases of severe intrusions, the strategy and importance multidisciplinary approach comprising surgery, orthodontics, and endodontics to allow exposure of the crown, orthodontic extrusion, and onset of endodontic therapy [10]. In the present case, an alteration of convenience form for root canal access was performed via buccal face making easier and faster this fundamental operatory step.

Endodontic treatment was initiated 15 days after the accident. In accordance with Tronstad et al. [13], it was stated that root canal therapy for intruded teeth with open apices must be started 1-2 weeks after occurrence of trauma. In order to ensure the need of a radical root canal treatment, vitality pulpal test was performed using Endo-Ice, by means of a frozen cotton pellet. No sensitivity was related by the patient in both teeth. Due to the possibility of false negative for such kind of test, the operators performed the access without anesthesia, and only for tooth 21, the patient related a mild sensitivity during this step. The choice of mepivacaine 3% without vasoconstrictor was important to avoid the influence of constriction of pulp vessels (a transient ischemia). Despite this procedure, for both teeth, the pulps were found necrotic (ischemic and without bleeding).

The use of a root canal dressing with a material based on calcium hydroxide (Calen) between sessions was aimed at dissolving remaining pulpal debris, alkalinizing the environment preventing inflammatory root resorption [18].

Cvek [19] reached 96% of success in cases of apexification with long-term treatment with calcium hydroxide. The author reported that its alkaline pH and physical presence inside the root canal represent an effective antibacterial effect by inhibiting osteoclastic activity, avoiding the penetration of granulation and exsudate tissue and by forming a hard tissue barrier.

Despite 97% of all inflammatory resorptions are arrested after long-term calcium hydroxide treatment, there is no effective treatment for replacement resorption. The root is gradually resorbed leading eventually to loss of the tooth [2].

Replacement resorption also appears to be more frequent in mature than in immature teeth [20].

Presently, the indication of MTA in one visit is an alternative procedure for apexification treatment without intermediary dressings. This procedure has presented satisfactory results in other management of traumatized teeth with open apex [19]. 

Mineral trioxide aggregate (MTA) was introduced on the market as a new alternative method for apexification because this material is composed by several minerals including calcium oxide. This definitive material has good physico-chemical properties, present biocompatibility, and can be applied in a wet environment.

Due to chosen multidisciplinary approach, the role of orthodontic treatment was re-positioning of the injured teeth by means of segmented arch technique rather than continuous arch technique. This approach led to force magnitude (light and continuous) and direction control allowing the desired and precise movement.

Despite the satisfactory results, the use of mineral trioxide aggregate led to a mild crown discoloration, resulting in a grey aspect after clinical treatment with white MTA, fact already noticed in other two previous articles [21, 22]. These authors highlighted this adverse effect of MTA, even when using a white material. This detail cannot be disregarded, as most of the avulsed teeth involve the patient appearance and aesthetics.

## References

[1] Andreasen JO (1970). Luxation of permanent teeth due to trauma. A clinical and radiographic follow-up study of 189 injured teeth. *Scandinavian Journal of Dental Research*.

[2] Wigen TI, Agnalt R, Jacobsen I (2008). Intrusive luxation of permanent incisors in Norwegians aged 6–17 years: a retrospective study of treatment and outcome. *Dental Traumatology*.

[3] Andreasen FM (1989). Pulpal healing after luxation injuries and root fracture in the permanent dentition. *Endodontics &amp; Dental Traumatology*.

[4] Nelson-Filho P, Faria G, Assed S, Pardini LC (2006). Surgical repositioning of traumatically intruded permanent incisor: case report with a 10-year follow up. *Dental Traumatology*.

[5] Royal College of Surgeons of England Treatment of avulsed permanent teeth in children. http://www.rcseng.ac.uk/fds/clinical_guidelines.

[6] Andreasen JO (1972). *Traumatic Injuries to the Teeth*.

[7] Isaacson RJ, Rebellato J (1995). Two-couple orthodontic appliance systems: torquing arches. *Seminars in Orthodontics*.

[8] Shroff B (2001). Root correction during orthodontic therapy. *Seminars in Orthodontics*.

[9] Andreasen JO, Bakland LK, Matras RC, Andreasen FM (2006). Traumatic intrusion of permanent teeth—part 1. An epidemiological study of 216 intruded permanent teeth. *Dental Traumatology*.

[10] De Alencar AHG, Lustosa-Pereira A, De Sousa HA, Figueiredo JH (2007). Intrusive luxation: a case report. *Dental Traumatology*.

[11] Andreasen JO, Andreasen FM (2000). *Essentials of Traumatic Injuries to the Teeth: A Step-by-Step Treatment Guide*.

[12] Bruszt P (1958). Secondary eruption of teeth introduced intro the maxilla by a blow. *Oral Surgery, Oral Medicine, Oral Pathology*.

[13] Tronstad L, Trope M, Bank M, Barnett F (1986). Surgical access for endodontic treatment of intruded teeth. *Endodontics &amp; Dental Traumatology*.

[14] Araújo MAM, Valera MC (1999). *Tratamento Clínico dos Traumatismos Dentários*.

[15] Oulis C, Vadiakas G, Siskos G (1996). Management of intrusive luxation injuries. *Endodontics and Dental Traumatology*.

[16] Andreasen FM, Vestergaard PB (1985). Prognosis of luxated permanent teeth—the development of pulp necrosis. *Endodontics & Dental Traumatology*.

[17] Caliskan MK, Cinsar A, Türkün M, Akkemik Ö (1997). Delayed endodontic and orthodontic treatment of cross-bite occurring after luxuation injury in permanent incisor teeth. *Endodontics & Dental Traumatology*.

[18] Siqueira JF, Lopes HP (2004). *Endodontia—Biologia e Técnica*.

[19] Cvek M (1972). Treatment of non-vital permanent incisors with calcium hydroxide. I. Follow-up of periapical repair and apical closure of immature roots. *Odontologisk Revy*.

[20] Andreasen FM (1995). *Pulpal healing after luxation injuries and root fracture in the permanent dentition*.

[21] Jacobovitz M, De Pontes Lima RK (2009). The use of calcium hydroxide and mineral trioxide aggregate on apexification of a replanted tooth: a case report. *Dental Traumatology*.

[22] Jacobovitz M, De Lima RKP (2008). Treatment of inflammatory internal root resorption with mineral trioxide aggregate: a case report. *International Endodontic Journal*.

